# Functional identification of the zebrafish Interleukin-1 receptor in an embryonic model of Il-1β-induced systemic inflammation

**DOI:** 10.3389/fimmu.2022.1039161

**Published:** 2022-10-26

**Authors:** Dylan J. Sebo, Audrey R. Fetsko, Kallie K. Phipps, Michael R. Taylor

**Affiliations:** ^1^ Division of Pharmaceutical Sciences, School of Pharmacy, University of Wisconsin–Madison, Madison, WI, United States; ^2^ Pharmacology and Toxicology Program, School of Pharmacy, University of Wisconsin–Madison, Madison, WI, United States

**Keywords:** zebrafish, interleukin - 1 β, interleukin 1 receptor type 1, inflammation, neutrophils, ROS - reactive oxygen species, UAS/Gal4

## Abstract

Interleukin-1β (IL-1β) is a potent proinflammatory cytokine that plays a vital role in the innate immune system. To observe the innate immune response *in vivo*, several transgenic zebrafish lines have been developed to model IL-1β-induced inflammation and to visualize immune cell migration and proliferation in real time. However, our understanding of the IL-1β response in zebrafish is limited due to an incomplete genome annotation and a lack of functional data for the cytokine receptors involved in the inflammatory process. Here, we use a combination of database mining, genetic analyses, and functional assays to identify zebrafish Interleukin-1 receptor, type 1 (Il1r1). We identified putative zebrafish *il1r1* candidate genes that encode proteins with predicted structures similar to human IL1R1. To examine functionality of these candidates, we designed highly effective morpholinos to disrupt gene expression in a zebrafish model of embryonic Il-1β-induced systemic inflammation. In this double transgenic model, *ubb:Gal4-EcR*, *uas:il1β^mat^*, the zebrafish *ubiquitin b* (*ubb*) promoter drives expression of the modified Gal4 transcription factor fused to the ecdysone receptor (EcR), which in turn drives the tightly-regulated expression and secretion of mature Il-1β only in the presence of the ecdysone analog tebufenozide (Teb). Application of Teb to *ubb:Gal4-EcR*, *uas:il1β^mat^* embryos causes premature death, fin degradation, substantial neutrophil expansion, and generation of reactive oxygen species (ROS). To rescue these deleterious phenotypes, we injected *ubb:Gal4-EcR*, *uas:il1β^mat^* embryos with putative *il1r1* morpholinos and found that knockdown of only one candidate gene prevented the adverse effects caused by Il-1β. Mosaic knockout of *il1r1* using the CRISPR/Cas9 system phenocopied these results. Taken together, our study identifies the functional zebrafish Il1r1 utilizing a genetic model of Il-1β-induced inflammation and provides valuable new insights to study inflammatory conditions specifically driven by Il-1β or related to Il1r1 function in zebrafish.

## Introduction

Zebrafish (*Danio rerio*) have emerged as a valuable vertebrate model to study inflammation due to several important features including rapid development of the innate immune system, optical transparency for *in vivo* imaging, and the accessibility of genetic tools and experimental methods (1–3). The relative ease of transgenesis in zebrafish (4) has given rise to several genetic models of inflammation driven by the expression of the potent proinflammatory cytokine Interleukin-1β (Il-1β). These models include heat-shock-inducible Il-1β (5), cell-specific expression of Il-1β (6), and a doxycycline-inducible model (7). A variety of non-genetic zebrafish inflammation models have also been characterized, including infection models using bacteria (8–10), viruses (11), fungi (12, 13), and lipopolysaccharide (LPS) (14) as well as several wound-induced models (15–20). Similar to the genetic models, wound-induced models stimulate Il-1β expression and promote Il-1β dependent migration of neutrophils to the site of injury (5, 21). Additionally, morpholino knockdown of zebrafish Il-1β decreases the recruitment of neutrophils demonstrating that Il-1β plays a specific role in regulating neutrophil migration during injury-induced inflammation (5). More recently, we generated a genetically inducible model of systemic inflammation using the Gal4-EcR/UAS system (22). In this model, the mature form of Il-1β is secreted in response to the ecdysone analog tebufenozide resulting in dose-dependent neutrophil expansion, reactive oxygen species (ROS) generation, morbidity, and mortality (23).

During an innate immune response to foreign antigens, IL-1β is produced as an inactive precursor that is processed by caspase-1 and the inflammasome to produce mature IL-1β for secretion (24). Once secreted, IL-1β (as well as IL-1α) binds specifically to the Interleukin-1 receptor, type 1 (IL1R1) causing a conformational change that allows for binding of the co-receptor Interleukin-1 receptor accessory protein (IL1RAP; also known as IL1R3) that forms a trimeric complex that promotes a strong proinflammatory signal (25). To demonstrate functionality, previous studies showed that targeted disruption of the murine genes encoding either IL1R1 (26, 27) or IL1RAP (28) block IL-1 signaling, indicating that IL1R1 and IL1RAP are both required to elicit an immune response. While IL1R1 and IL1RAP share some structural similarities, both receptors possess unique primary sequences and distinct genomic locations in both human and mouse. As IL1R1 binds specifically to IL-1β and serves as a primary therapeutic target for treating inflammation in a broad spectrum of diseases, our current study focuses on the identification of the zebrafish Interleukin-1 receptor, type 1 (Il1r1).

Given the prominent role of Il-1β in the inflammatory response, it is particularly surprising that the zebrafish Il1r1 has not been identified. Indeed, the discovery and validation of functional orthologs in zebrafish can be challenging for a variety of reasons. While comparison of the human and zebrafish reference genomes reveals that approximately 70% of human genes have at least one obvious zebrafish ortholog, the sequence divergence of many genes is so great that they cannot be recognized or confirmed as orthologs without experimental validation (29). Furthermore, members of the Teleostei infraclass, including zebrafish, underwent a teleost-specific whole-genome duplication event (30). This further complicates the identification of human - zebrafish orthologs due to the possibility of 1) functionally redundant paralogs, 2) mutually exclusive, overlapping, or redundant expression patterns of the paralogs, or 3) the presence of pseudogenes.

In this study, we perform an *in silico* analysis and identify putative zebrafish Il1r1 orthologs with low sequence identities but with predicted protein structures highly similar to human IL1R1. To determine if these proteins function as the receptor for Il-1β, we utilize our validated *in vivo* zebrafish model of Il-1β-induced systemic inflammation (23). Here, we knockdown the expression of putative receptors and examine the phenotypic rescue of inflammation by performing multiple functional assays. Using this experimental approach, we identify the functional ortholog for zebrafish Il1r1 and demonstrate that it is absolutely required for the Il-1β-driven inflammatory response and the associated disease phenotypes. Given the increasing interest in zebrafish models of inflammation, our findings provide valuable new tools to dissect signaling pathways, cellular responses, and disease models that are specifically driven by or related to Il1r1 function.

## Materials and methods

### Zebrafish husbandry

Zebrafish lines were maintained using guidelines established in The Zebrafish Book (31). The AB wild-type (WT) strain was acquired from the Zebrafish International Resource Center. Transgenic lines *Tg(ubb:IVS2GVEcR, cmcl2:EGFP)*, herein abbreviated as *ubb:Gal4-EcR*, and *Tg(uas:GSP-Il1β^mat^, cmlc2:mCherry)*, herein abbreviated as *uas:Il1β^mat^* were previously generated in our lab (23). The *Tg(mpx:mCherry)^uwm7^* transgenic line, herein abbreviated as *mpx:mCherry*, was a gift from Dr. Anna Huttenlocher (UW-Madison). Embryos were maintained at 28.5°C in egg water (0.03% Instant Ocean reconstituted in reverse osmosis water). For imaging, 0.003% phenylthiourea (PTU) was used to inhibit melanin production. All experiments were performed in accordance with the University of Wisconsin-Madison Institutional Animal Care and Use Committee.

### Database mining for putative zebrafish interleukin-1 receptors

To identify putative zebrafish Interleukin-1 receptor(s), type 1 (Il1r1), we performed database searches for zebrafish homologs. The human IL1R1 protein sequence (accession number P14778) was used to search the zebrafish genome at Ensembl (assembly GRCz11) using the TBLASTN search tool. Sequence identities and phylogenetic analysis of protein sequences was performed using the Clustal V Method in MegAlign (DNASTAR). Predicted protein structures for human IL1R1 (https://alphafold.ebi.ac.uk/entry/P14778), zebrafish CABZ01054965 (https://alphafold.ebi.ac.uk/entry/E7FGC6), and zebrafish ZMP:0000000936 (https://alphafold.ebi.ac.uk/entry/E7F5V6) were identified using AlphaFold (32, 33).

### Morpholino antisense oligonucleotide design and microinjection

Morpholino antisense oligonucleotides (MO) were designed against zebrafish *cabz01054965* and *zmp:0000000936* using the manufacturers recommendations (GeneTools). The *cabz01054965* splice donor morpholino sequence 5’-TGTGCATCAGGGTTTACCTTTCGC-3’ was designed against the splice donor site (underlined) located between exon 4 and intron 5. The *zmp:0000000936* splice donor morpholino sequence 5’-GTGATATGAAAGGCTCACCCTGCAC-3’ was designed against the splice donor site (underlined) located between exon 5 and intron 6. The *zmp:0000000936* start site morpholino sequence 5’-ACCAATCGACCCATATCTACAGCCG-3’ was designed to span across the translation start site (underlined). For control morpholino injections, the standard GeneTools control oligo was used 5’-CCTCTTACCTCAGTTACAATTTATA-3’. Uninjected embryos were used as controls for some experiments as indicated.

Morpholinos were resuspended in dH_2_O at a stock concentration of 2 mM. For microinjection, morpholinos were diluted to 0.1-0.5 mM in dH_2_O containing phenol red (0.05%) as an injection tracer. Microinjection needles were fabricated from 1.2 mm thin wall glass capillaries (WPI; TW120F-4) using a Sutter Instrument Flaming/Brown Micropipette Puller (Model P-97). Approximately 2 nl were microinjected into the yolk of single-celled embryos from pair-wise crosses of the relevant genotypes. Damaged embryos identified with gross morphological defects as a result of microinjection were removed from the study prior to analyses. All morpholinos were found to be effective at a concentration of 0.25 mM (~2 ng) without any obvious off-target effects.

### Generation of Il-1β-induced embryonic systemic inflammation

The transgenic lines *ubb:Gal4-EcR* and *uas:Il1β^mat^* were used to generate Il-1β-induced embryonic systemic inflammation in the presence of the ecdysone analog, tebufenozide (Teb), as previously described (23). Teb was prepared as a 1 mM stock solution in 100% DMSO. Adults carrying the transgenes were bred, embryos were injected with morpholino at the one-cell stage or left uninjected, and then treated at 1 or 2 days postfertilization (dpf) with 1 µM Teb or 0.1% DMSO (untreated controls). Embryos were selected for both transgenesis markers *cmlc2:EGFP* (green heart) and *cmlc2:mCherry* (red heart) using a Nikon SMZ18 epifluorescence stereomicroscope prior to further analyses. For neutrophil experiments, the *ubb:Gal4-EcR* and *uas:Il1β^mat^* lines were bred to *mpx:mCherry*. All experimental embryos were heterozygous for all transgenes.

### Mosiac rescue of Il-1β-induced embryonic systemic inflammation with CRISPR/Cas9

To phenocopy the *zmp* splice-donor and start-site morpholino rescue of Il-1β-induced embryonic systemic inflammation, we used a CRISPR/Cas9 strategy utilizing the crRNA:tracrRNA duplex format with recombinant *S. Pyogenes* Cas9 nuclease (Cas9) from Integrated DNA Technologies (IDT). Using the Alt-R Custom Cas9 crRNA Design Tool (IDT), we designed two CRISPR RNAs (crRNAs): cr1.*zmp:0000000936*.ex8 5’-/AltR1/ucgacugcuggacaccagacguuuuagagcuaugcu/AltR2/-3’and cr2.*zmp:0000000936*.ex9 5’-/AltR1/uuaagguggagcuggucuuaguuuuagagcuaugcu/AltR2/-3’ against exon 8 and exon 9, respectively. For CRISPR-Cas9 ribonucleoprotein (RNP) preparation and microinjection, we followed the IDT demonstrated protocol “Zebrafish embryo microinjection” modified from Dr. Jeffrey Essner (Iowa State University). The crRNAs and transactivating crRNA (tracrRNA) were resuspended to 100 µM in nuclease-free TE buffer, pH 8.0, the individual crRNAs were combined with tracrRNA at 1:1 molar ratio in nuclease-free Duplex Buffer (IDT) to create 3 µM gRNA complexes (cr1 and cr2). The solutions were heated to 95°C for 5 min, then cooled to room temperature. Recombinant Cas9, glycerol-free (IDT) was diluted to 0.5 µg/µl in PBS, pH 7.4. The RNP complex was assembled by combining 3 µl of gRNA complexes with 3 µl of diluted Cas9, incubated at 37°C for 10 min, then cooled to room temperature. Approximately 2 nl of the RNP complexes with either cr1, cr2, or combined cr1/cr2 (1:1) was microinjected into *ubb:Gal4-EcR*, *uas:Il1β^mat^* single-cell embryos and monitored for morbidity and mortality. PCR was performed on individual embryos using *zmp:0000000936*-specific primers: forward primer 5’-tatgtgttcctcttgcagCG-3’ and reverse primer 5’-tgtttatacgagcacCTGTGG-3’ located in intron 7 and intron 9, respectively. Percentages of survival (alive), morbidity (sick), and mortality (dead) were plotted for each group using Excel (Microsoft). To test for differences between groups, a chi-square test of independence was performed, followed by a *post hoc* test using adjusted residuals and a p-value Bonferroni adjustment.

### RNA extraction, cDNA synthesis, and RT-PCR

Embryos from 0-3 dpf (20-30 per group in duplicate) were anesthetized in 0.02% Tricaine, transferred into RNase/DNase-free 1.5 ml microcentrifuge tubes with fitted pestle (Kontes), homogenized in TRIzol, and total RNA was extracted according to the manufacturer’s protocol (Invitrogen). cDNA was synthesized by reverse transcription using the SuperScript IV First-Strand Synthesis System using Oligo(dT) primers according to the manufacturer’s protocol (Invitrogen). Reverse transcription polymerase chain reaction (RT-PCR) amplified a 578 bp fragment of *cabz01054965* using forward primer 5’-ACGCACCTGACACATCGTAA-3’ and reverse primer 5’-GTTTGACTTGGCTTCGGGTA-3’, a 577 bp fragment of *zmp:0000000936* using forward primer 5’-GCGAGATGACCTCAGAAACC-3’ and reverse primer 5’-TCCTCCGACACATGAGACAC-3’, and a 932 bp fragment of *actin, beta 1* (*actb1*) as an RT-PCR control using forward primer 5’-CCCTCCATTGTTGGACGAC-3’ and reverse primer 5’-CCGATCCAGACGGAGTATTTG -3’. All primers were designed using Primer3 (34).

### Whole-mount *in situ* hybridization

WISH was performed using protocols adapted from Vauti et al. and Thisse and Thisse (35, 36). This new protocol afforded greater probe penetration into the central nervous system (CNS) and trunk of 1-3 dpf animals used for this study. Briefly, WT embryos from 1-3 dpf were fixed in 4% paraformaldehyde/PBS at 4°C overnight, dehydrated in 100% methanol, then stored at −20°C. For probe synthesis, *cabz01054965 (forward primer* 5’-*AATTAACCCTCACTAAAGGG*GCGAGATGACCTCAGAAACC-3’; reverse primer 5’-*TAATACGACTCACTATAGGG*ACCTCCTCCTCCTCTTCCAG-3’) and *zmp:0000000936 (forward primer* 5’-*AATTAACCCTCACTAAAGGG*GCGAGATGACCTCAGAAACC-3’; reverse primer 5’-*TAATACGACTCACTATAGGG*ACCTCCTCCTCCTCTTCCAG-3’) were PCR amplified from WT zebrafish cDNA to produce products of 1,227 and 1,293 bp, respectively. Both forward primers contained a 5’-T3 RNA polymerase binding site (*italics*) and both reverse primers contained a 5’-T7 RNA polymerase binding site (*italics*). PCR products were then purified using the QIAquick PCR purification kit (Qiagen). Approximately 1 µg of purified PCR product was used to synthesize sense and antisense digoxigenin (DIG)-labeled RNA probes with T3 and T7 RNA polymerase, respectively, using a DIG RNA Labeling Kit (Roche). Prepared embryos (n=8 per group) were transitioned to ethanol, treated with ethanol/xylol (1:1 vol/vol), rehydrated in H_2_O with 0.1% Tween, permeabilized in 80% Acetone, and bleached in H_2_O_2_, according to Vauti et al., 2020 (36). Embryos were prehybridized at 70°C for 2 hr, then hybridized with DIG-labeled RNA probes at 70°C overnight. After washing and blocking, the embryos were incubated with anti-DIG-AP Fab fragment (Roche) at 4°C overnight then stained with BM Purple, AP precipitating substrate (Roche), containing NBT and BCIP, until the desired signal intensity appeared (35). Images were captured using a Nikon SMZ18 stereomicroscope equipped with a Nikon DS-Fi2 color camera and Nikon NIS-Elements software.

### Survival analysis

To examine Il-1β-induced mortality, double transgenic *ubb:Gal4-EcR*, *uas:Il1β^mat^* embryos were induced with 1 µM Teb at 1 dpf and survival was monitored until 6 dpf. All survival assays were performed in 100x15 mm petri dishes (Falcon). In our previous survival studies, Teb (concentration range: 10 nM-10 µM) was added at 2 dpf, which caused mortality beginning at 5 dpf (23). Therefore, in the current study, we added Teb at 1 dpf to expedite mortality as morpholino effectiveness may be reduced past 3 dpf (37). Survival was tallied daily as dead embryos were identified and removed from the petri dishes. Teb was replaced daily with freshly prepared solution. Kaplan-Meier curves were made using Excel (Microsoft) and log rank tests were used to evaluate statistical significance.

### Gross morphology

Transgenic *ubb:Gal4-EcR* and *uas:Il1β^mat^* embryos (10-20 per group) were induced with 1 µM Teb at 2 dpf and maintained in fresh Teb until 4 dpf. Three embryos per group (except for *cabz* +Teb; n=2), were randomly selected and imaged. Lateral images were acquired by confocal microscopy using the TD channel at 4 dpf. Regions of interest were quantified using FIJI ROI selection and measurement tools. Mean cross sectional area + standard deviation was plotted using Excel (Microsoft).

### Neutrophil quantification

The neutrophil reporter line *mpx:mCherry* was bred to *ubb:Gal4-EcR*, *uas:Il1β^mat^* double transgenics to produce triple heterozygous transgenic embryos. Systemic inflammation was induced at 2 dpf with 1 µM Teb (10-20 per group). Three embryos per group (except for *cabz* +Teb; n=2), were randomly selected and neutrophils were imaged by confocal microscopy at 4 dpf. Neutrophils were quantified by manually counting the number of mCherry-positive cells throughout the entire volume rendered image using FIJI Cell Counter Plugin (38). Mean + standard deviation was plotted using Excel (Microsoft).

### Reactive oxygen species analysis

Transgenic *ubb:Gal4-EcR*, *uas:Il1β^mat^* embryos (10-20 per group) were induced with 1 µM Teb at 2 dpf and maintained in Teb until 3 dpf. CM-H_2_DCFDA (Invitrogen), a fluorescent cell-permeant indicator for ROS, was freshly prepared as a 10 mM stock in DMSO. Embryos were incubated with 2.5 μM CM-H_2_DCFDA (Invitrogen) for 30 min in the dark, then washed in egg water (3 x 10 minutes each wash) prior to imaging. Three embryos per group (except for *cabz* +Teb; n=2), were randomly selected and imaged by confocal microscopy at 3 dpf. Quantification of fluorescent signal was completed on 2D projections of 3D confocal z-stacks, created using the Nikon NIS-Elements Maximum Intensity Projection algorithm. Regions of interest were identified using FIJI ROI selection tools, then the fluorescence was quantified using the FIJI ‘mean grey value’ measurement tool. Values were normalized to uninjected no Teb controls and normalized mean + standard deviation was plotted using Excel (Microsoft).

### Confocal laser scanning microscopy

Zebrafish from 1 to 4 dpf were anesthetized in 0.02% Tricaine and immobilized in 1.2% low melting point agarose (Invitrogen) in glass bottom culture dishes (MatTek). Confocal microscopy was performed using a Nikon Eclipse Ti microscope equipped with a Nikon A1R. For images of whole embryos and larvae, large images (4 x 1 mm) were captured and stitched together with a 15% overlap. All images are 2D projections of 3D confocal z-stacks using the Nikon NIS-Elements Maximum Intensity Projection algorithm or are single frame lateral TD images. All image manipulation for brightness or contrast (*via* Nikon NIS-Elements software) was applied to all pixels, equally, and does not affect interpretation of data.

### Statistical analysis

Statistical differences of mean values among multiple groups were determined using one-way analysis of variance (ANOVA) followed by Tukey’s HSD *post-hoc* test. The criterion for statistical significance was set at *P* < 0.05. Values represent means ± standard deviation. Error bars show +1 standard deviation.

## Results

### Identification of the putative zebrafish interleukin-1 receptor, type 1

**Figure 1:**
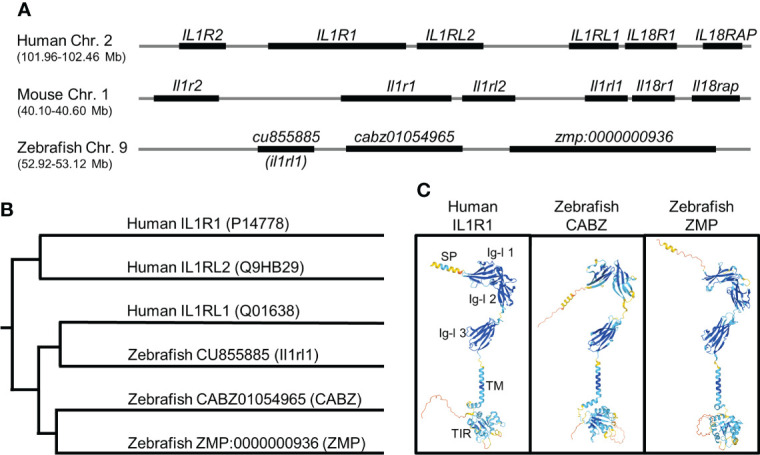
*In silico* identification of the putative zebrafish interleukin-1 receptor, type 1 (Il1r1). **(A)** Alignment of human, mouse, and zebrafish genomic regions showing conserved synteny. Shown here are the human and mouse IL-1 receptor clusters and the putative zebrafish IL-1 receptor cluster (not to scale). **(B)** Phylogenetic alignment of human IL1R protein sequences with putative zebrafish Il1r protein sequences. **(C)** Predicted protein structures of human IL1R1, zebrafish CABZ (CABZ01054965), and zebrafish ZMP (ZMP:0000000936) showing conserved domains: N-terminal signal peptide (SP), 3 extracellular immunoglobulin-like (Ig-l) domains (Ig-l 1, Ig-l 2, and Ig-l 3), a single transmembrane domain (TM), and an intracellular Toll/Interleukin-1 receptor (TIR).

Using the human IL1R1 protein sequence (accession number: P14778), we performed a TBLASTN against the zebrafish genome at Ensembl. We identified three genes, *cabz01054965*, (accession number: ENSDARG00000090844), *zmp:0000000936* (accession number: ENSDARG00000088672), and *cu855885*, (accession number: ENSDARG00000101527) on zebrafish chromosome (chr.) 9 with sequence similarities to human IL1R1. In addition, these three genes showed partial synteny to the IL-1 Receptor Cluster on human chr. 2q (39) and mouse chr. 1 (**Figure 1A**). The *cu855885* gene has previously been identified as the zebrafish ortholog for human Interleukin-1 receptor-like 1 (*IL1RL1*) (40), herein referred to as zebrafish *il1rl1*. Protein sequence alignment using the Clustal V Method in MegAlign (DNASTAR) indicated percent identities to the human IL1R1 protein sequence of 19.6%, 21.4%, and 19.1% for the proteins encoded by the zebrafish genes *cabz01054965*, *zmp:0000000936*, and *il1rl1*, respectively.

Phylogenetic analysis of human IL1R1 (P14778), human IL1RL1 (Q01638), human IL1RL2 (Q9HB29), zebrafish CABZ01054965, zebrafish ZMP:0000000936, and zebrafish Il1rl1 was also performed using the Clustal V Method in MegAlign (**Figure 1B**). As predicted, zebrafish Il1rl1 was found to be most similar to human IL1RL1, confirming the previously established annotation (40). However, our analysis was unable to definitively distinguish whether CABZ01054965 or ZMP:0000000936 were functional zebrafish orthologs or paralogs for either human IL1R1 or IL1RL2.

We next examined the structural similarities between human IL1R1, zebrafish CABZ01054965 (abbreviated herein as CABZ), and ZMP:0000000936 (abbreviated herein as ZMP) using the AlphaFold Protein Structure Database (32, 33). As shown in **Figure 1C**, human IL1R1 consists of an N-terminal signal peptide (SP), 3 extracellular immunoglobulin-like (Ig-l) domains, a single transmembrane domain (TM), and an intracellular Toll/Interleukin-1 receptor (TIR) domain (41–43). Despite the low sequence similarities between the human and zebrafish proteins, AlphaFold predicted conserved protein structures and domains for human IL1R1, zebrafish CABZ, and zebrafish ZMP (**Figure 1C**). However, these structures did not distinguish functionality between the two zebrafish proteins.

We also examined the zebrafish genome for *IL1RAP* and the three additional *IL1R* genes found within the human and mouse IL-1 receptor clusters (i.e. *IL1R2*, *IL18R1*, and *IL18RAP*). As IL-1 signaling requires the association of IL1RAP with the IL-1β/IL1R1 complex, we mined the zebrafish genome for the gene encoding the zebrafish ortholog for Il1rap. BLAST analysis using human IL1RAP localized the putative zebrafish *il1rap* gene (*cabz01068246*) to chr. 15 near the zebrafish *fgf12b* gene (data not shown). This genomic region in zebrafish shares conserved synteny with both the human and mouse genomes, which show colocalization of the *IL1RAP* and *FGF12* genes. In addition, BLAST analysis using human IL1R2 localized the putative zebrafish *il1r2* (*cabz01078737*) near the proximal telomere of chr. 9 adjacent to zebrafish *map4K4* (data not shown). This genomic region shows conserved synteny between *il1r2* and *map4K4* in human, mouse, and zebrafish, albeit at a distant genomic region in the zebrafish genome, with respect to the zebrafish IL-1 receptor cluster. No obvious zebrafish orthologs for human or mouse IL18R1 or IL18RAP were identified using BLAST analysis. However, the previously annotated zebrafish gene products Il1rapl1a, Il1rapl1b, and Il1rapl2 (44) share partial sequence alignment to human and mouse IL18R1 and IL18RAP, indicating the potential for conserved function.

### Developmental expression of putative zebrafish interleukin-1 receptors

**Figure 2:**
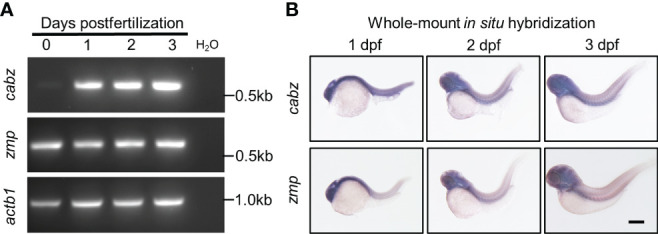
Developmental expression of putative zebrafish *Il1r1*. **(A)** RT-PCR from WT embryos at 0-3 dpf showed expression of *cabz* beginning at 1 dpf, maternal expression of *zmp* at 0 dpf, and *actin beta 1* (*actb1*) transcript as an experimental control. Molecular weight markers are shown on the right. **(B)** Whole-mount *in situ* hybridization using antisense DIG-labeled riboprobes against *cabz* or *zmp* transcripts showed unrestricted expression of both transcripts at 1, 2, and 3 dpf. Scale bar in **(B)** is 200 µm.

To examine the developmental expression of the zebrafish *cabz* and *zmp* transcripts, we performed reverse-transcription polymerase chain reaction (RT-PCR) and whole-mount *in situ* hybridization (WISH). No antibodies against the zebrafish proteins are currently available for protein expression analysis, and no expression data for either zebrafish transcript is currently available. For RT-PCR, we extracted total RNA from embryos at 0, 1, 2, and 3 days postfertilization (dpf), synthesized cDNA, and PCR amplified using sequence-specific DNA primers (see Materials and Methods). As shown in **Figure 2A**, the *cabz* transcript was not detected maternally, whereas the *zmp* transcript demonstrated maternally derived expression at 0 dpf. Both the *cabz* and *zmp* transcripts showed robust expression from 1-3 dpf, indicating that both transcripts are developmentally expressed (**Figure 2A**).

To examine the spatiotemporal expression of the zebrafish *cabz* and *zmp* transcripts, we performed WISH. Using wild-type (WT) embryos at 1, 2, and 3 dpf, we analyzed transcript expression using antisense and sense digoxigenin (DIG)-labeled RNA probes. As expected, sense probes did not detect any expression (data not shown). We found that the *cabz* and *zmp* antisense probes exhibited unrestricted expression of both transcripts at all three developmental stages examined (**Figure 2B**). The unrestricted expression of these zebrafish *il1r* genes is consistent with low tissue specificity of the human *IL1R1* transcript as demonstrated by RNAseq, microarray, and SAGE analysis (see https://www.proteinatlas.org/ENSG00000115594-IL1R1/tissue and https://www.genecards.org/cgi-bin/carddisp.pl?gene=IL1R1#expression).

### Morpholino knockdown of the putative zebrafish interleukin-1 receptors

**Figure 3:**
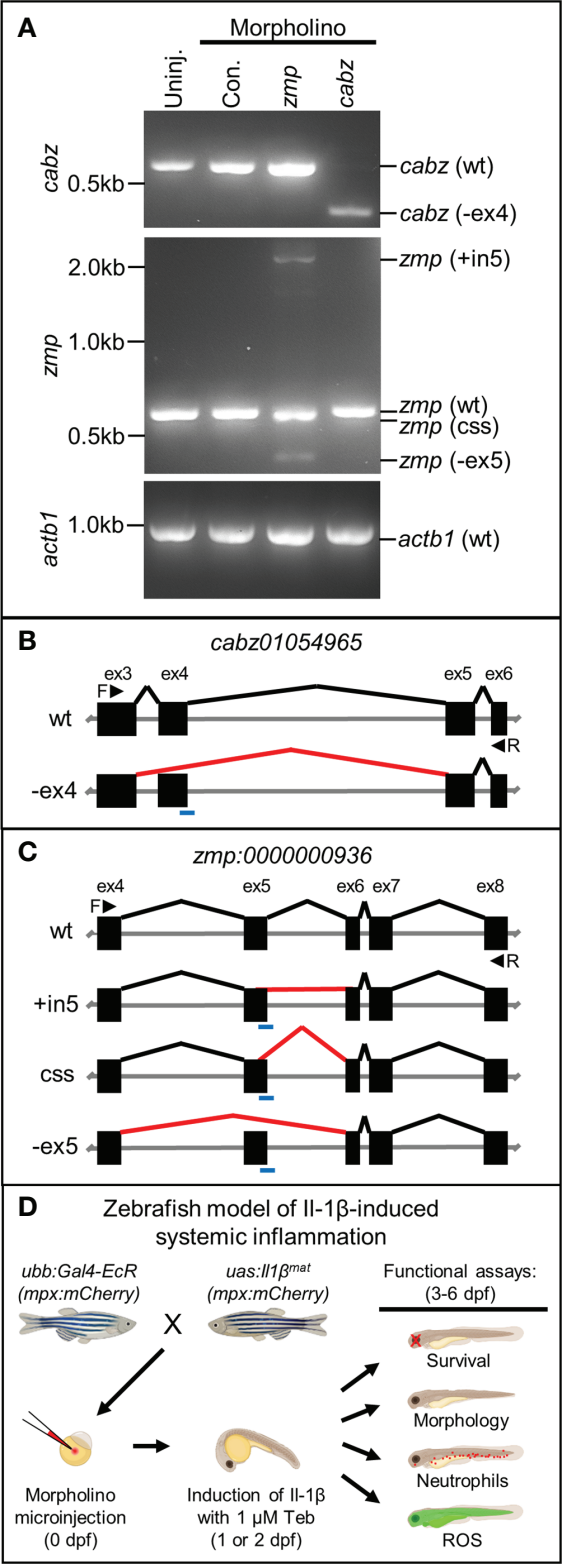
Morpholino knockdown of putative zebrafish *Il1r1*. **(A)** RT-PCR of *cabz*, *zmp*, and *actb1* transcripts from uninjected embryos, control morpholino injected embryos, and *cabz* and *zmp* morphants. **(B)** Schematic of the *cabz01054965* (*cabz*) gene showing exons 3-6, forward (F) and reverse (R) primers used for analysis (black arrowheads), the location of the splice donor site morpholino (blue line), normal splicing of wt (black lines), and aberrant splicing with the *cabz* morpholino (red line) that excludes exon 4 (-ex4). **(C)** Schematic of the *zmp:0000000936* (*zmp*) gene showing exons 4-8, forward (F) and reverse (R) primers used for analysis (black arrowheads), the location of the splice donor site morpholino (blue line), normal splicing of wt (black lines), and aberrant splicing with the *zmp* morpholino (red lines) that includes intron 5 (+in5), introduce a cryptic splice sites (css), or excludes exon 5 (-ex5). **(D)** Diagram of the experimental paradigm used to functionally identify zebrafish Il1r1. A zebrafish model of Il-1β-induced systemic inflammation was generated by breeding transgenic lines *ubb:Gal4-EcR* and *uas:Ilβ^mat^* with or without the neutrophil reporter line *mpx:mCherry*. Single-celled embryos were injected with morpholino, inflammation was induced with 1 µM Tebufenozide (Teb) at 1 or 2 dpf, and functional analyses were performed from 3-6 dpf to assess morpholino rescue of Il-1β-induced inflammation.

Based upon sequence homology and transcript expression results, we were unable to conclusively identify the functional homolog(s) for zebrafish Il1r1. Therefore, we designed morpholino oligonucleotides (MO) against splice donor sites to functionally knockdown the expression of both CABZ and ZMP by disrupting pre-mRNA splicing (45). Morpholinos were injected into WT embryos at the single-cell stage. The resulting morphants were raised to 2 dpf, total RNA was extracted, then morphant transcripts were analyzed by RT-PCR using DNA primers designed to span the splice donor sites of *cabz* and *zmp*, or the *actin beta 1* (*actb1*) transcript as a control. We found that injection of the *cabz* splice donor morpholino resulted in the loss of the WT transcript (578 bp) and the formation of a single alternatively spliced transcript of approximately 400 bp (**Figure 3A**; top panel). In addition, we found that injection of the *zmp* splice donor morpholino resulted in the loss of the WT transcript (577 bp) and the formation of three aberrantly spliced transcripts of approximately 2,100, 570, and 400 bp (**Figure 3A**; middle panel). As expected, the standard control morpholino (Con.) supplied by GeneTools showed no effect on the *zmp*, *cabz*, or *actb1* transcripts. To further examine the splicing effects of both morpholinos, we gel purified and sequenced the aberrantly spliced transcripts. For the *cabz* morphants, we found that the ~400 bp band was due to exclusion of exon 4 (-ex4), which was predicted to result in a non-functional product (**Figures 3A** **, B**). For the *zmp* morphants, we found that the ~2,100 bp band was due to the inclusion of intron 5 (+in5), the ~570 bp band was due to a cryptic splicing site (css) in exon 5 twelve bases upstream of the authentic splice-donor site, and the ~400 bp band was due to exclusion of exon 5 (-ex5) (**Figures 3A** **, C**). All three alternatively spliced *zmp* morphant transcripts were predicted to result in non-functional products. Importantly, the ~570 bp band from the *zmp* splice-site morphants is slightly smaller than the WT band and no WT transcript was detected in the *zmp* splice-site morphants using DNA sequence analysis. In fact, no WT transcripts were detected in either of the *cabz* or *zmp* morphants at the developmental stages examined. Thus, both the *cabz* and *zmp* morpholinos provide valuable genetic tools that disrupt the expression of these transcripts.

To test the functional consequences of the *cabz* and *zmp* morpholinos, we utilized our transgenic zebrafish model of Il-1β-induced systemic inflammation (23). We previously generated two transgenic zebrafish lines *Tg(ubb:IVS2GVEcR, cmcl2:EGFP)*, herein abbreviated as *ubb:Gal4-EcR*, and *Tg(uas:GSP-Il1β^mat^, cmlc2:mCherry)*, herein abbreviated as *uas:Il1β^mat^*. This model uses the Gal4-EcR/UAS system (22), where mature Il-1β (Il1β^mat^) is ubiquitously secreted only in the presence of the ecdysone analog, Tebufenozide (Teb). In order to identify the functional Il1r1 in zebrafish, we used our model to examine 1) survival, 2) morphology, 3) neutrophils (*mpx:mCherry*), and 4) reactive oxygen species (ROS) in the putative Il1r1 morphants (**Figure 3D**).

### Survival analysis of Il-1β-induced mortality

**Figure 4:**
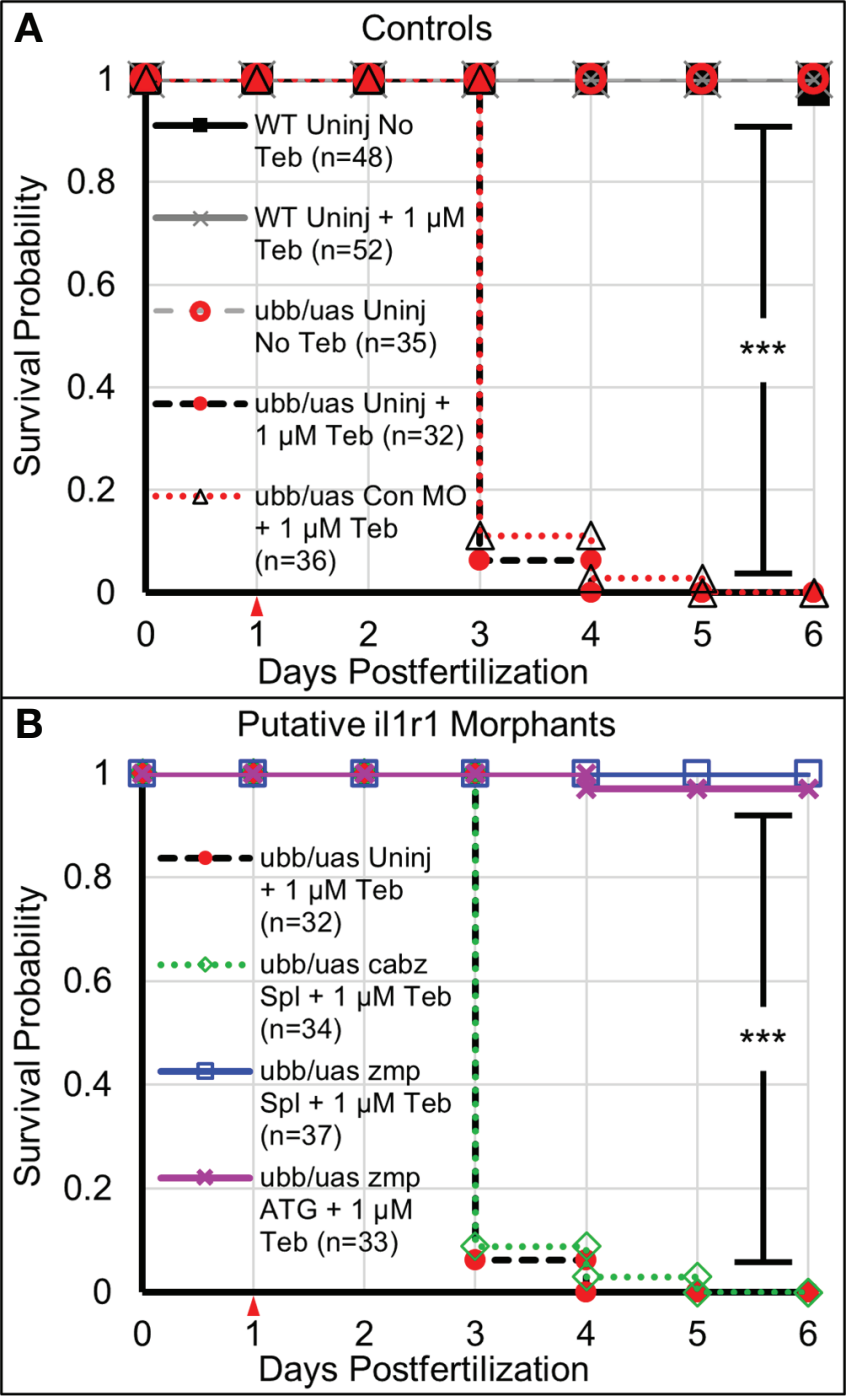
Survival analysis of Il-1β-induced mortality. **(A)** Kaplan-Meier representations of the survival of WT larvae left untreated (WT Uninj No Teb), or Teb treated (WT Uninj + 1 µM Teb), *ubb:Gal4-EcR*, *uas:Ilβ^mat^* larvae with no Teb treatment (ubb/uas Uninj No Teb), with Teb (ubb/uas Uninj + 1 µM Teb), or injected with control morpholino with Teb (ubb/uas Con MO + 1 µM Teb). **(B)** Kaplan-Meier representations of the survival of *ubb:Gal4-EcR*, *uas:Ilβ^mat^* larvae left uninjected with teb treatment (ubb/uas Uninj + 1 µM Teb) injected with the cabz splice donor morpholino with Teb (ubb/uas cabz Spl + 1 µM Teb), zmp splice donor morpholino with Teb (ubb/uas zmp Spl + 1 µ M Teb), or zmp start site morpholino with Teb (ubb/uas zmp ATG + 1 µM Teb). Teb was added to the embryos at 1 dpf (red arrowhead). Note that only the *zmp* morpholinos rescued Il-1β-induced death. ***p<0.001.

Our previous studies established Il-1β-induced mortality in double transgenic *ubb:Gal4-EcR*, *uas:Il1β^mat^* larvae in the presence of Teb in a dose-dependent manner (23). To accelerate mortality in our current study, we modified the original experimental paradigm by generating inflammation at 1 dpf, which coincides with the onset of innate immunity in zebrafish (10, 46, 47). Here, double transgenic *ubb:Gal4-EcR*, *uas:Il1β^mat^* embryos were 1) injected with control, *cabz*, or *zmp* morpholinos at the single-cell stage or left uninjected, 2) treated with 1 µM Teb or no Teb (0.1% DMSO) at 1 dpf, and then 3) observed daily for survival up to 6 dpf. As shown in **Figure 4A**, all untreated *ubb:Gal4-EcR*, *uas:Il1β^mat^* controls (ubb/uas Uninj No Teb) and all wild type controls (WT Uninj No Teb and WT Uninj + 1 µM Teb) survived to 6 dpf and beyond. Moreover, none of the morpholino injections or Teb treatments significantly reduced survival in WT embryos at the concentrations used (**Supplemental Figure 1**). In contrast, when *ubb:Gal4-EcR*, *uas:Il1β^mat^* embryos were treated with 1 µM Teb, the majority of uninjected controls (ubb/uas Uninj + 1 µM Teb; *n*=32) and control morphants (ubb/uas Con MO + 1 µM Teb; *n*=36) died by 3 dpf (**Figure 4A**). As control MO showed no effect on morbidity and mortality, the remainder of controlled experiments were conducted with uninjected embryos only. These results demonstrate that Teb-treated *ubb:Gal4-EcR*, *uas:Il1β^mat^* embryos are extremely susceptible to mortality and that this experimental paradigm provides a high level of reproducibility.

Concurrently, we monitored survival of the putative *il1r1* morphants. We reasoned that if Il-1β-induced mortality is mediated by the activation of zebrafish Il1r1, then morpholino knockdown of the appropriate receptor(s) would reduce death and increase survival. As shown in **Figure 4B**, we found that the *cabz* splice-site morphants treated with 1 µM Teb (ubb/uas cabz Spl + 1 µM Teb; *n*=34) died at a rate comparable to the uninjected and control MO injected Teb treated embryos, indicating that Il-1β-induced death is not mediated through zebrafish CABZ. In contrast, the *zmp* splice-site morphants treated with 1 µM Teb (ubb/uas zmp Spl + 1 µM Teb; *n*=37) survived equally to No Teb embryos (**Figures 4A** **, B**). To confirm the effects of the *zmp* splice-site morpholino, we designed and injected a *zmp* start-site morpholino (*zmp* ATG) to block translation of the *zmp* transcript (48). As with the *zmp* splice morphants, the *zmp* start morphants (ubb/uas zmp ATG + 1 µM Teb; *n*=33) survived at a frequency comparable to No Teb embryos (**Figures 4A** **, B**).

To further verify and phenocopy the *zmp* morpholino rescue of Il-1β-induced morbidity and mortality, we microinjected CRISPR-Cas9 ribonucleoprotein (RNP) complexes targeting the *zmp* gene into *ubb:Gal4-EcR*, *uas:Il1β^mat^* embryos to generate mosaic knockouts. Two RNPs, cr1 and cr2, targeting exon 8 and exon 9, respectively, were injected independently or in combination at 1:1 molar ratio (**Supplemental Figure 2A**) then treated with 1 µM Teb (+ Teb) or untreated (No Teb). As shown in **Supplemental Figure 2B**, uninjected control embryos (Con.) treated with Teb at 1 dpf showed ~80% dead, ~15% sick (fin degradation and general morbidity), and ~5% alive by 4 dpf. In contrast, the RNP-injected embryos showed a dramatic increase in survival with ~85% of cr1/cr2, ~94% of cr1, and ~50% of cr2 alive at 4 dpf. Injection of RNPs had no effect on survival in the absence of Teb treatment. As cr1/cr2 injection could result in a deletion mutation, we analyzed crispants by PCR. We found that the controls, cr1, and cr2 produced a predicted 508 bp band, whereas the cr1/cr2 combination resulted in the 508 bp band as well as a smaller ~160 bp band in three of the four samples shown (**Supplemental Figure 2C**). As the protospacer adjacent motif (PAM) site of cr1 and cr2 are ~350 bp apart, this smaller band likely represents a genomic deletion between cr1 and cr2. We also conclude that the cr1 and cr2 crispants must cause in a small inactivating insertion or deletion (indel) in *il1r1* as both rescued Il-1β-induced mortality.

Our data demonstrate that knockdown of zebrafish ZMP function by disrupting pre-mRNA splicing, blocking translation, or mosaic CRISPR/Cas9 knockout rescues Il-1β-dependent mortality. Since both *zmp* splice-site and *zmp* start-site morpholinos equally rescued death and worked as effectively as mosaic knockout with CRISPR/Cas9, we used the splice-site morpholinos for the remainder of experiments described below.

### Rescue of Il-1β-induced gross morphological defects

**Figure 5:**
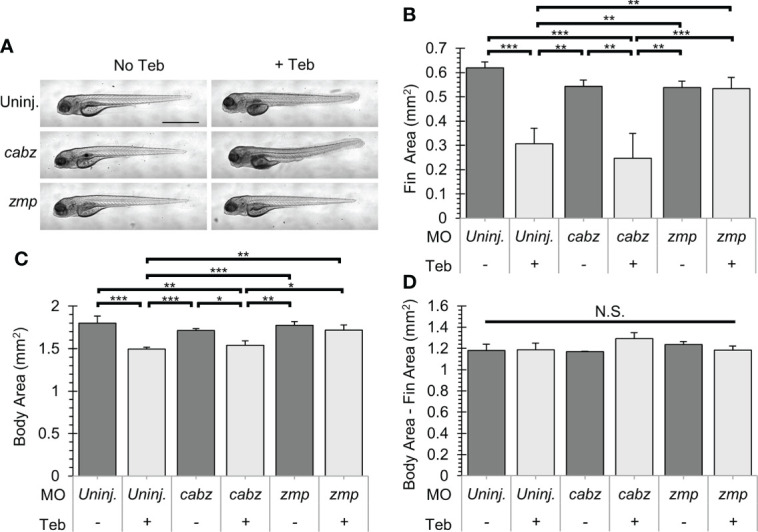
Gross morphological analysis of Il-1β-induced morbidity. **(A)** Representative images of *ubb:Gal4-EcR*, *uas:Ilβ^mat^* larvae showing gross morphology. Shown here are uninjected larvae, *cabz* morphants, and *zmp* morphants without Teb (No Teb) and with Teb treatment (+Teb). Note that *zmp* morphants +Teb showed normal morphology with no gross defects. **(B–D)** Mean Cross-Sectional Fin Area **(B)**, Body Area **(C)**, and the product of Body Area – Fin Area **(D)** was plotted in mm^2^ for each group. Error Bars are +1 standard deviation. Asterisks indicate significant differences *p < 0.05 **p < 0.01 ***p < 0.001; N.S., not significant; by one-way ANOVA followed by Tukey’s HSD Test. Scale bar in **(A)** is 1 mm.

Double transgenic *ubb:Gal4-EcR*, *uas:Il1β^mat^* embryos were 1) uninjected, injected with *cabz* morpholino (*cabz*), or injected with *zmp* morpholino (*zmp*), 2) untreated (No Teb) or treated with 1 µM Teb at 2 dpf, and then 3) imaged for gross morphology at 4 dpf. We previously demonstrated that Il-1β-induced systemic inflammation results in fin degradation and general morbidity prior to death (23). As shown in **Figure 5A**, untreated larvae (No Teb) maintained normal morphology, whereas Teb-treated larvae (+Teb) showed extensive fin degradation and an overall unhealthy phenotype (**Figure 5A**; top panels). Untreated *cabz* morphants also displayed normal fin morphology, whereas Teb-treated embryos showed extensive fin degradation similar to the uninjected controls (**Figure 5A**; middle panels). In contrast, *zmp* morphants displayed normal fin morphology with and without Teb treatment, indicating rescue of the inflammation phenotype (**Figure 5A**; bottom panels). Furthermore, quantification of fin degradation revealed that the *zmp* morpholino, but not the *cabz* morpholino, prevented Il-1β-dependent morbidity in our model. We found that the total fin area was significantly decreased with Teb treatment in the uninjected and the *cabz* morphants, whereas the *zmp* morphants maintained normal fin area similar to untreated larvae (**Figure 5B**). We also measured the effects on total body area between the different groups. We found that Teb-induced larvae had a smaller cross-sectional area compared to untreated controls (**Figure 5C**). However, all cross-sectional area differences were a consequence of fin degradation only (**Figure 5D**). No other gross morphological differences were observed between the groups. In addition, Teb-treatment (even up to 10 µM Teb) showed no effect on morphology in WT embryos without the *ubb:Gal4-EcR*, *uas:Il1β^mat^* transgenes (**Supplemental Figure 3**).

### Inhibition of Il-1β-induced neutrophil expansion

**Figure 6:**
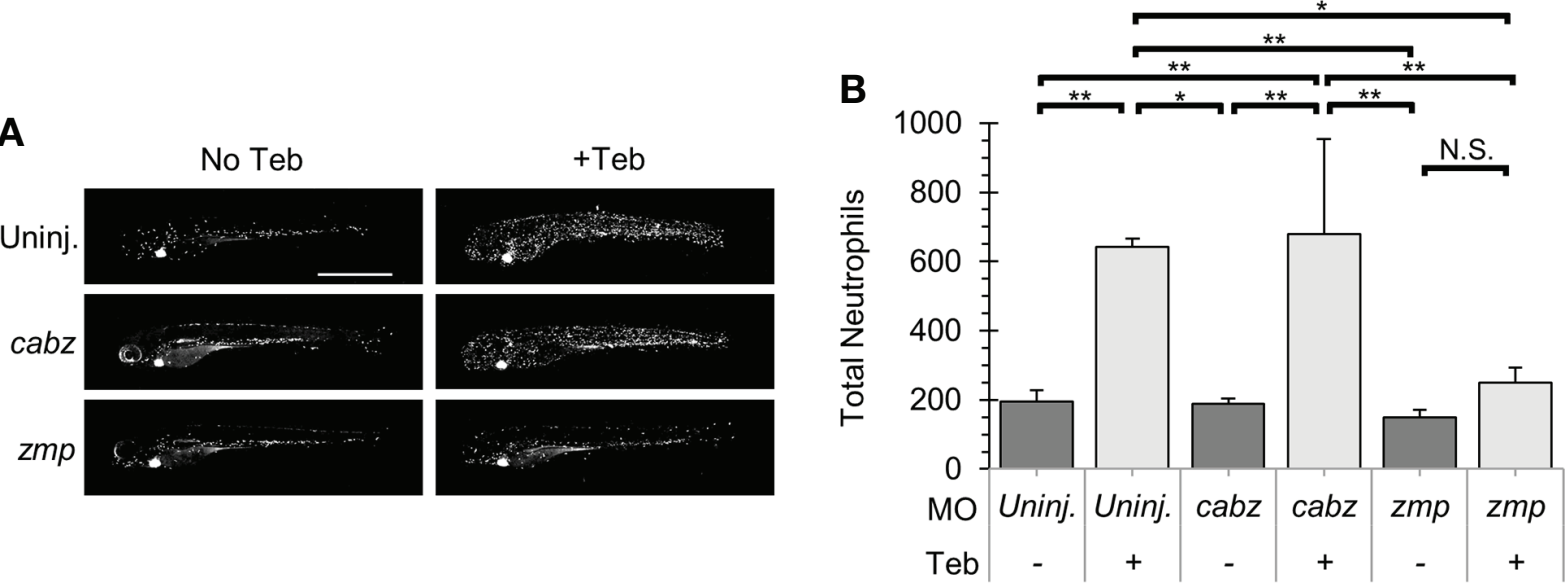
Il-1β-induced neutrophil recruitment. **(A)** Representative confocal images of *ubb:Gal4-EcR*, *uas:Ilβ^mat^, mpx:mCherry* larvae. Shown here are uninjected larvae, *cabz* morphants, and *zmp* morphants without Teb (No Teb) and with Teb treatment (+Teb). **(B)** Neutrophils were counted using FIJI Cell Counter and Total Neutrophil counts were plotted for each group. Error Bars are +1 standard deviation. Asterisks indicate significant differences *p < 0.05 **p < 0.01; N.S., not significant; by one-way ANOVA followed by Tukey’s HSD Test. Scale bar in **(A)** is 1 mm.

Neutrophils are innate immune cells responsive to proinflammatory cytokines such at Il-1β. In zebrafish, neutrophils become functional during early development (10, 46), after which they can expand and migrate in response to inflammatory events such as tissue damage or microbial infection (18, 49). Here, we investigated whether our putative *il1r1* morpholinos were able to block Il-1β-dependent neutrophil activity. Triple transgenic *ubb:Gal4-EcR*, *uas:Il1β^mat^*, *mpx:mCherry* embryos were 1) uninjected, injected with *cabz* morpholino (*cabz*), or injected with *zmp* morpholino (*zmp*), 2) untreated (No Teb) or treated with 1 µM Teb at 2 dpf, and then 3) imaged and quantified at 4 dpf. As shown in **Figure 6A**, untreated larvae (No Teb), showed a stereotypical distribution of neutrophils primarily located in the caudal hematopoietic tissue (CHT) with few cells dispersed throughout the larvae (left panels). When treated with Teb (+Teb), uninjected larvae and *cabz* morphants showed an extensive expansion of neutrophils within the CHT and throughout the larvae, whereas *zmp* morphants were phenotypically similar to untreated (No Teb) larvae. Teb showed no effects on neutrophil numbers or distribution in *mpx:mCherry* embryos (**Supplemental Figure 4**). To quantify these results, we counted total neutrophils in the whole larvae (**Figure 6B**). In uninjected larvae and *cabz* morphants, Teb treatment caused a significant increase in the total number of neutrophils. In contrast, the Teb-treated *zmp* morphants showed no significant neutrophil expansion, indicating complete phenotypic rescue by the *zmp* morpholino.

### Prevention of Il-1β-induced reactive oxygen species

**Figure 7:**
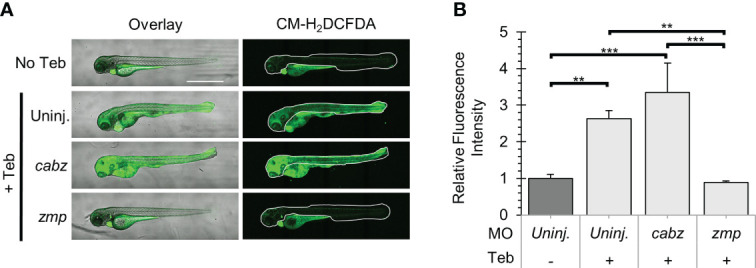
Analysis of Reactive Oxygen Species in Il-1β-induced inflammation. **(A)** Representative confocal images of *ubb:Gal4-EcR*, *uas:Ilβ^mat^* larvae treated with CM-H_2_DCFDA. Shown here are the overlay of brightfield and CM-H_2_DCFDA fluorescence (left panels) and CM-H_2_DCFDA fluorescence only (right panels). Fluorescence signal was quantified from the region of interest outlined with a white border in the CM-H_2_DCFDA images. **(B)** Relative Fluorescence Intensity (RFI) was quantified using FIJI ‘mean grey value’ measurement. All mean RFIs were normalized to uninjected No Teb mean values. Error Bars are +1 standard deviation. Asterisks indicate significant differences **p < 0.01 ***p < 0.001 by one-way ANOVA followed by Tukey’s HSD Test. Scale bar in **(A)** is 1 mm.

ROS are central to the progression of many inflammatory diseases and are produced by cells, such as neutrophils, that are involved in the host-defense response to various cytokines (50). For this study, we examined whether our putative *il1r1* morpholinos were capable of blocking Il-1β-dependent ROS production. Double transgenic *ubb:Gal4-EcR*, *uas:Il1β^mat^* embryos were 1) uninjected, injected with *cabz* morpholino (*cabz*), or injected with *zmp* morpholino (*zmp*), 2) untreated (No Teb) or treated with 1 µM Teb at 2 dpf, 3) exposed to CM-H_2_DCFDA, a fluorescent cell-permeant indicator for ROS, at 4 dpf, and 4) imaged and quantified for relative fluorescence. Here, we show an overlay of brightfield and fluorescent images (**Figure 7A**; left panels) and the area of fluorescence quantified (**Figure 7A**; right panels, white boundaries). As untreated larvae showed fluorescence in the yolk, gut, and heart from autofluorescence, CM-H_2_DCFDA cleavage, and *cmlc2:EGFP*, respectively, we excluded these regions from quantification. To quantify the signal, we measured the fluorescence within the white boundaries using FIJI Mean Gray Value Measurement as described in Methods. As shown in **Figure 7B**, addition of Teb caused a significant increase in the fluorescent signal in the uninjected controls, demonstrating Il-1β-dependent ROS production. Similarly, the *cabz* morphants showed a dramatic increase in ROS, indicating that the *cabz* morpholino did not block this process. In contrast, the *zmp* morphants showed almost no fluorescent signal, equivalent to the uninjected larvae without Teb. These data indicate that the *zmp* morpholino blocks Il-1β-dependent ROS production.

Given that 1) the *zmp* gene shares conserved synteny between the human, mouse, and zebrafish genomes, 2) *zmp* morpholinos rescued mortality, gross morphology, neutrophil counts, and prevented ROS in our model of Il-1β-induced embryonic systemic inflammation, and 3) mosaic CRISPR/Cas9 knockout phenocopies the morpholino rescue, we have genetically and functionally demonstrated that the *zmp:0000000936* gene encodes zebrafish Il1r1.

## Discussion

In this study, we use a combination of database mining, genetic analyses, and functional assays to identify zebrafish Il1r1. Although our *in silico* analysis revealed several putative genes that encode for proteins with partial alignment to human IL1R1, we discovered two putative candidate genes with conserved synteny to the human and mouse genomes. The proteins encoded by these genes show relatively low sequence identities to human IL1R1 but have highly similar predicted protein structures. To examine functionality of these candidates, we designed highly effective morpholinos that disrupted normal splicing or transcription with no obvious off-target effects. We found that knockdown of only one candidate rescued neutrophil expansion, ROS generation, morbidity, and mortality in our zebrafish model of embryonic Il-1β-induced systemic inflammation. We also designed effective gRNAs for mosaic knockout using the CRISPR/Cas9 system that phenocopied the knockdown effects of the morpholinos. We therefore conclude that the zebrafish genome contains only one functional ortholog of Il1r1. Our study highlights the importance of functional data to accurately identify and annotate zebrafish orthologs of inflammation genes and provides essential insights to the inflammatory response driven by Il-1β.

As a potential caveat of our results, Il1rap is also required for IL-1 signaling and our study has not conclusively eliminated the possibility that *zmp:0000000936* could encode Il1rap. For example, knockdown of either zebrafish Il1r1 or Il1rap could both result in the rescue of Il-1β-induced inflammation as previously demonstrated by knockouts of either mouse Il1r1 or Il1rap (26–28). However, based upon conserved synteny and protein alignments, we predict that zebrafish Il1rap is most likely encoded by the *cabz01068246* gene (see Results), although we do not present functional data for this observation. In addition, by process of elimination, we predict that the *cabz01054965* gene, which is adjacent to *zmp:0000000936*, likely encodes the zebrafish Il1rl2 ortholog as shown by conserved synteny. Based upon our collective data, we are confident that we have identified zebrafish Il1r1, but have not conclusively demonstrated receptor functionality without a formal biochemical analysis of receptor/ligand binding.

Prior to our study, zebrafish Il1r1 was not accurately annotated in the zebrafish genome. Previous studies used the zebrafish genomic sequence to identify putative genes encoding Toll-like receptors (TLR) and interleukin receptors that contain a Toll/IL-1 receptor (TIR) domain. For example, Meijer et al. identified several predicted TLR proteins, one putative Il1r, and one putative Il18r (51). This study confirmed the expression of the predicted transcripts by RT-PCR and investigated their expression in *Mycobacterium*-infected zebrafish. Ultimately, the authors concluded that other *il1r* and *il18r* homologues may exist as the expression levels of the putative *il1r* and *il18r* transcripts were unaffected by *Mycobacterium* infection. On review of this work, we determined that the transcript-specific primers used to amplify *il1r* and *il18r* by RT-PCR are actually against *cu855885* (annotated as *il1rl1*) located on chr. 9 and *il18r* (annotated as *il1rap12*) located on chr. 14, respectively. More recently, Frame et al. described the identification of the zebrafish gene encoding Interleukin-1 receptor-like 1 (Il1rl1), a predicted homolog of zebrafish Il1r1. This study found upregulation of zebrafish *il1rl1* in Flk1^+^ cMyb^+^ hematopoietic stem and progenitor cells (HSPCs) in response to Il-1β stimulation by macrophages. The authors also found reduced *runx1*/*cmyb* expression and reduced numbers of CD41^+^ HSPCs following morpholino knockdown of *il1rl1* and suggest that Il-1β promotes Il1rl1^+^ HSPC production *via* inflammasome activity (40). Based upon the results we present in our study; it would be interesting to examine the expression and knockdown of zebrafish *il1r1* in this model of HSPC production.

To identify the functional zebrafish Il1r1, we implemented our validated transgenic model of embryonic Il-1β-induced systemic inflammation and used morpholino oligonucleotides to knockdown the expression of putative Il1r1 orthologs. In our model, the mature form of Il-1β is secreted only in the presence of the ecdysone analog tebufenozide (Teb), resulting in adverse inflammation-related phenotypes. Other inducible transgenic models, such as Tet-On/Tet-Off and Cre-ER recombinase often have unintended consequences due to antibiotic- and estrogen receptor-mediated effects that may complicate data interpretation (52). In contrast, we used the Teb-inducible UAS/Gal4-EcR system as there is no endogenous ecdysone receptor in vertebrates, no known off-target effects, and markedly low toxicity (22). Further, our inflammation model is driven by a single proinflammatory cytokine, Il-1β. Unlike wound-induced or infection-based models that cause inflammation *via* a myriad of damage-associated molecular patterns (DAMPs) and pathogen-associated molecular patterns (PAMPs), the use of a single molecular effector allowed for our targeted, morpholino-based approach. To demonstrate morpholino effectiveness in our model, we showed 1) disrupted pre-mRNA splicing as demonstrated by RT-PCR and DNA sequencing, 2) phenocopy of the splice-site morpholino using an independent *il1r1* start-site morpholino, 3) no obvious off-target phenotypes, and 4) complete rescue of the deleterious phenotypes caused by induced expression of Il-1β. Thus, our experimental paradigm, with appropriate caveats considered, follows the basic guidelines for morpholino use in zebrafish as described by Stainier et al. (53).

While newer technologies enable targeted gene disruption in zebrafish, including the CRISPR/Cas9 system, transcription activator-like effector nucleases (TALENs), and zinc finger nucleases (ZFNs) (54), these strategies require significant time and resources compared to validated, highly effective morpholinos. For example, the successful design and generation of CRISPR/Cas9 knockouts in combination with breeding schemes to produce double or triple transgenic zebrafish in a mutant background could take years to successfully accomplish. Furthermore, these analyses generally target one gene at a time, so if two paralogs were to have overlapping function, both genes would need to be targeted and bred into the appropriate background to observe the desired phenotypes. These analyses could be further complicated by compensatory networks that buffer against deleterious mutations (55). Given these limitations, we were surprised to find that mosaic CRISPR/Cas9 knockout of zebrafish Il1r1 effectively phenocopied the knockdown effects of the morpholinos. Thus, our study highlights the effective use of morpholinos and mosaic CRISPR/Cas9 to identify gene function in a relevant embryonic disease model simply by rescuing the deleterious phenotypes.

In conclusion, our study provides important new tools and insights for investigating the role of Il-1β and Il1r1 in a broad spectrum of diseases, such as autoinflammatory diseases, metabolic syndromes, acute and chronic inflammation, and malignancies (25, 56). As an example, neonatal-onset multisystem inflammatory disease (NOMID), a rare congenital inflammatory disorder, is caused by autosomal dominant mutations in the NLRP3 gene, also known as Cryopyrin or CIAS1 (57). Clinical manifestations in NOMID, similar to our zebrafish inflammation model, are caused by increased release of Il-1β. Current therapies for NOMID and related disorders primarily involve biologics against IL1R1 signaling such as Anakinra (a recombinant IL1R antagonist), Canakinumab (an antibody targeting Il-1β), and Rilonacept (a soluble decoy receptor). Given that many inflammatory conditions are driven by Il-1β and that there are no known small molecule inhibitors of IL1R1 (25, 56), identification of Il1r1 in our zebrafish model provides a novel platform to identify new drugs for the treatment of Il-1β-related diseases.

## Data availability statement

The original contributions presented in the study are included in the article/**Supplementary Material**. Further inquiries can be directed to the corresponding author.

## Ethics statement

The animal study was reviewed and approved by University of Wisconsin-Madison, Institutional Animal Care and Use Committee.

## Author contributions

DS conducted the experiments, collected, assembled, analyzed the data, and wrote the manuscript. AF provided experimental support, helped in data analysis, and edited the manuscript. KP provided experimental support and helped in data analysis. MT conceived the study, designed experiments, interpreted data, and wrote the manuscript. All authors contributed to the article and approved the submitted version.

## Funding

Funding was provided by NIH RO1NS116043. AF was supported by the UW-Madison Biotechnology Training Program, NIH T32GM008349 and T32GM135066.

## Acknowledgments

We thank Dr. Anna Huttenlocher (University of Wisconsin-Madison) for providing the *Tg(mpx:mCherry)^uwm7^* transgenic line. We also wish to thank Randall Kopielski and Jessica Fairbanks for assistance with zebrafish husbandry and animal facility care and maintenance.

## Conflict of interest

The authors declare that the research was conducted in the absence of any commercial or financial relationships that could be construed as a potential conflict of interest.

## Publisher’s note

All claims expressed in this article are solely those of the authors and do not necessarily represent those of their affiliated organizations, or those of the publisher, the editors and the reviewers. Any product that may be evaluated in this article, or claim that may be made by its manufacturer, is not guaranteed or endorsed by the publisher.
