# Supplementary material for: Functional identification of the zebrafish Interleukin-1 receptor in an embryonic model of Il-1β-induced systemic inflammation
Source: Front Immunol. 2022 Oct 26;13:1039161. doi: 10.3389/fimmu.2022.1039161 (PMC9643328; doi:10.3389/fimmu.2022.1039161)
Supplement: Supplementary file 1 [file DataSheet_1.pdf]

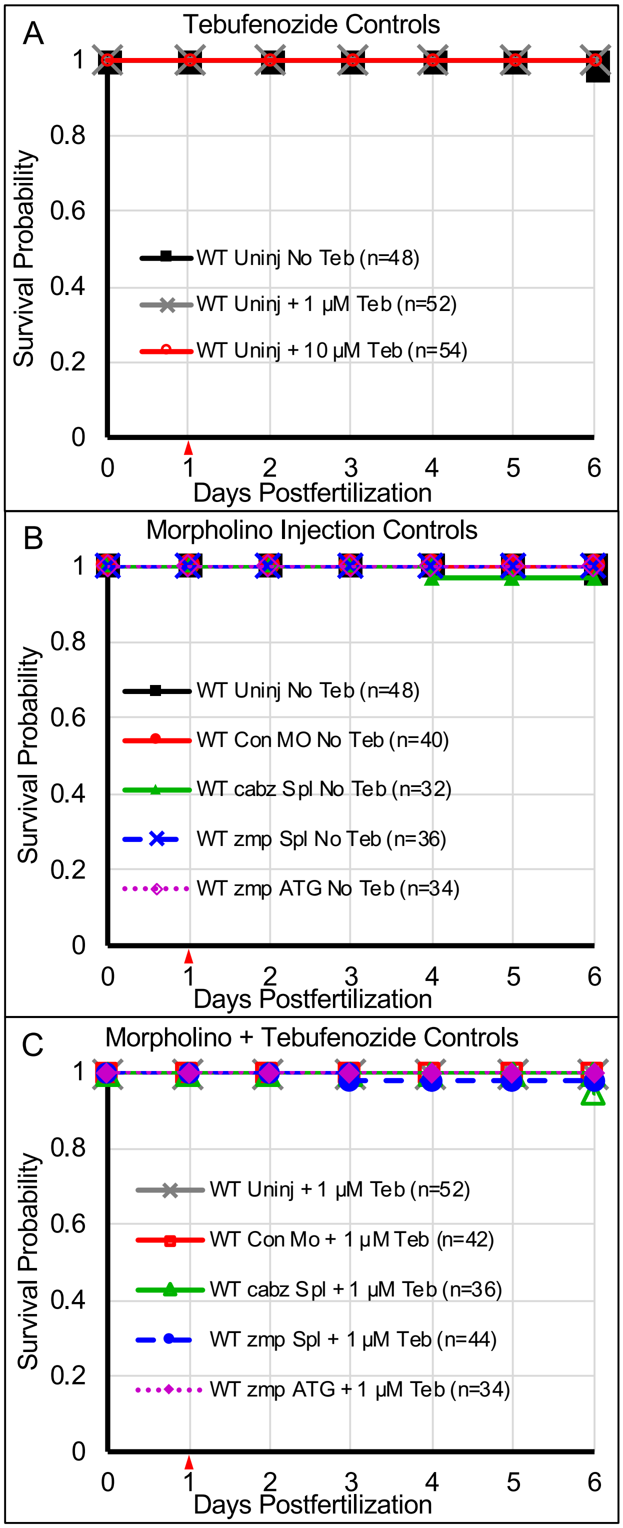

Supplemental Figure 1. Survival analysis of controls for Il-1β-induced mortality. **(A)** Kaplan-Meier representations of the survival of WT uninjected larvae with various Teb treatments as labeled. **(B)** Kaplan-Meier representations of the survival of WT larvae left uninjected or injected with control morpholino (Con MO), *cabz* splice donor morpholino (*cabz Spl*), *zmp* splice donor morpholino (*zmp Spl*), or *zmp* start site morpholino (*zmp ATG*), all without Teb Treatment. **(C)** Kaplan-Meier representations of the survival of WT larvae left uninjected or injected with control morpholino (Con MO), *cabz* splice donor morpholino (*cabz Spl*), *zmp* splice donor morpholino (*zmp Spl*), or *zmp* start site morpholino (*zmp ATG*), all with 1 µM Teb Treatment. Teb was added to the embryos at 1 dpf (red arrowheads). Note that no treatment or morpholino injection caused mortality in WT larvae.

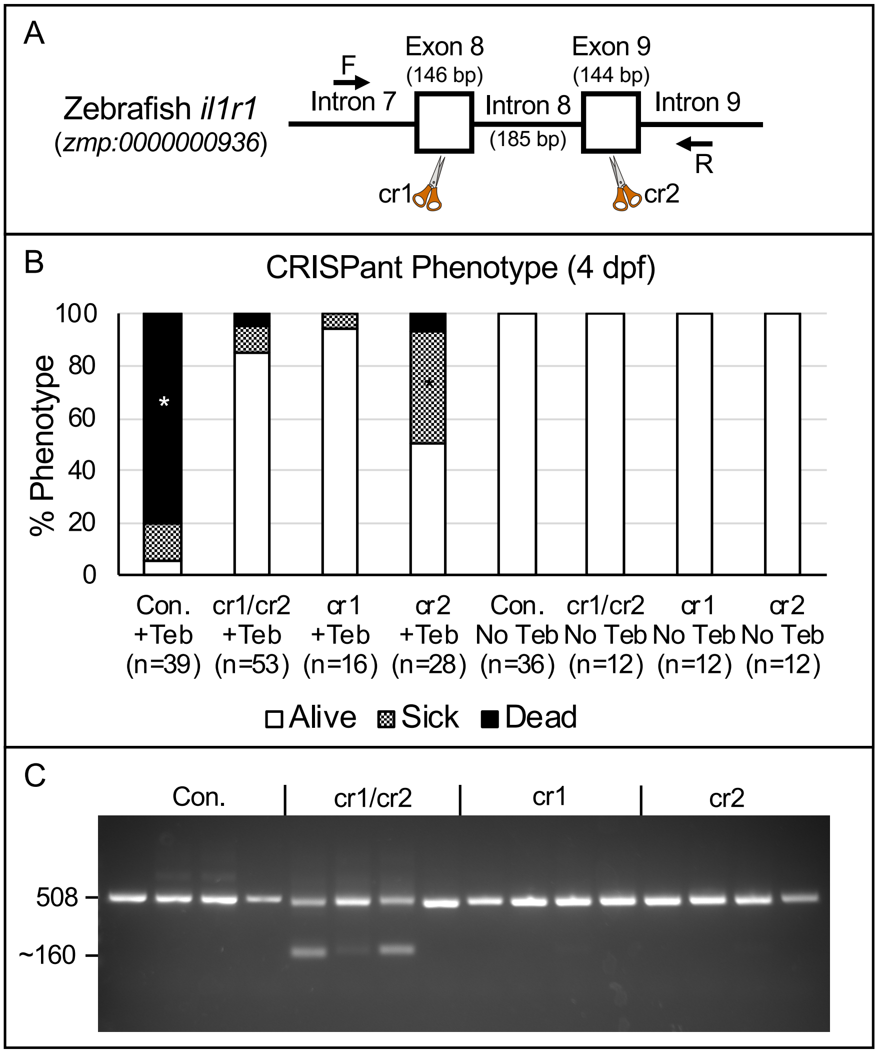

Supplemental Figure 2. Mosaic knockout of zebrafish *il1r1* (*zmp:0000000936*) using CRISPR/Cas9. **(A)** Two gRNAs, cr1 and cr2, were designed against exon 8 (cr1.*zmp:0000000936*.ex8) and exon 9 (cr2.*zmp:0000000936*.ex9), respectively. The gRNAs were combined with recombinant Cas9 to form a ribonuceoprotein complex and microinjected into *ubb:Gal4-EcR, uas:Il1β*^{mat} single-cell embryos either individually or in combination, untreated (No Teb) or treated with 1 μM Teb (+ Teb) at 1 dpf. **(B)** CRISPant phenotype was examined at 4 dpf and scored as Alive, Sick, or Dead. Note that CRISPants cr1 and cr1/cr2 treated with Teb survive normally with few sick or dead. In contrast, cr2 worked less efficiently when used alone. CRISPants cr1, cr2, and cr1/cr2 showed off-target effects when untreated with Teb. Asterisks (*) indicate statistically significant z-values of adjusted residuals as described in Materials and Methods. **(C)** PCR primers from intron 7 and intron 9 (see panel A) were used to amplify genomic DNA from individual CRISPants. Shown here are individual PCR samples from 4 controls, 4 cr1/cr2 coinjected, 4 cr1 only, and 4 cr2 only. Note that the controls, cr1 only, and cr2 only generate a single 508 bp product, whereas the cr1/cr2 samples generate a 508 bp and ~160 bp product indicating a genomic deletion.

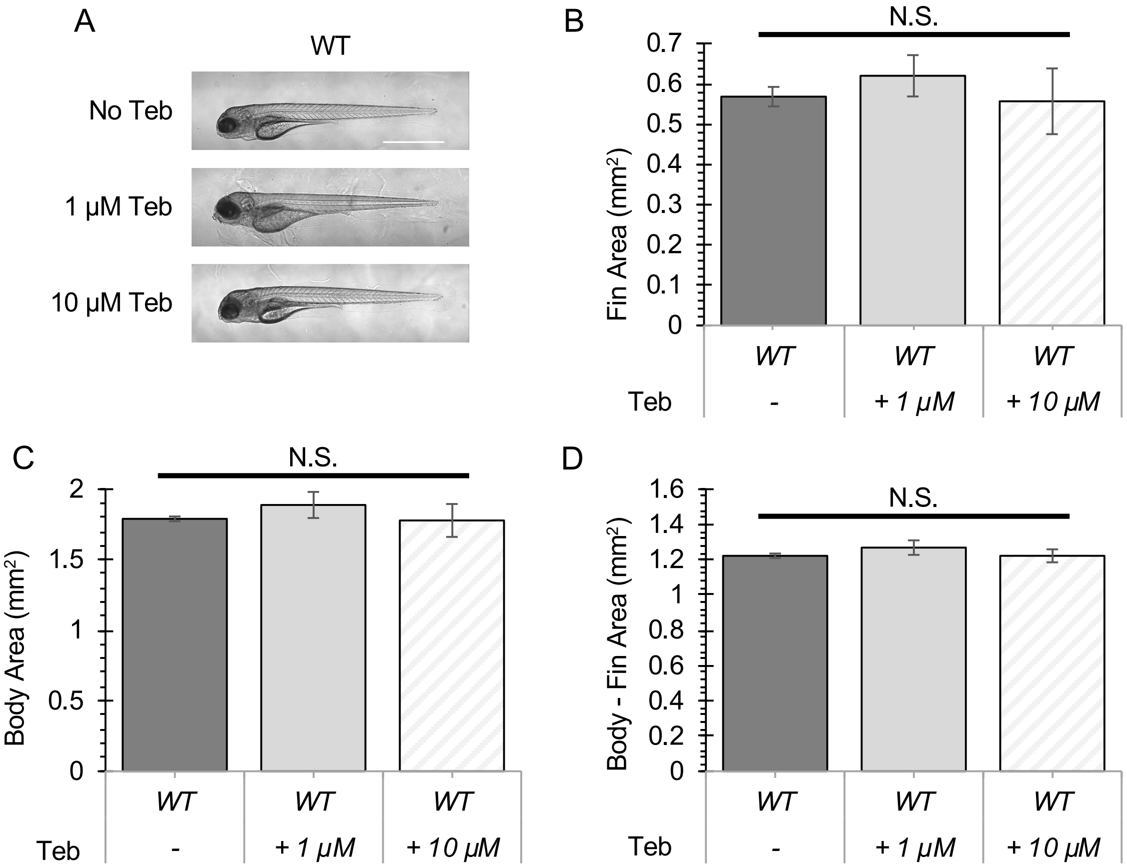

Supplemental Figure 3. Gross morphological analysis of controls for Il-1β-induced morbidity. **(A)** Representative images of WT larvae showing gross morphology. Shown here are larvae left untreated, treated with 1 μM Teb, or treated with 10 μM Teb. Note that all images showed normal morphology with no gross defects. **(B, C, D)** Mean Cross-Sectional Fin Area (**B**), Body Area (**C**), and the product of Body Area – Fin Area (**D**) was plotted in mm^2 for each group. Error Bars are +1 standard deviation. Asterisks indicate significant differences *p<0.05 **p<0.01 ***p<0.001; N.S., not significant; by one-way ANOVA followed by Tukey's HSD Test. Scale bar in (A) is 1 mm.

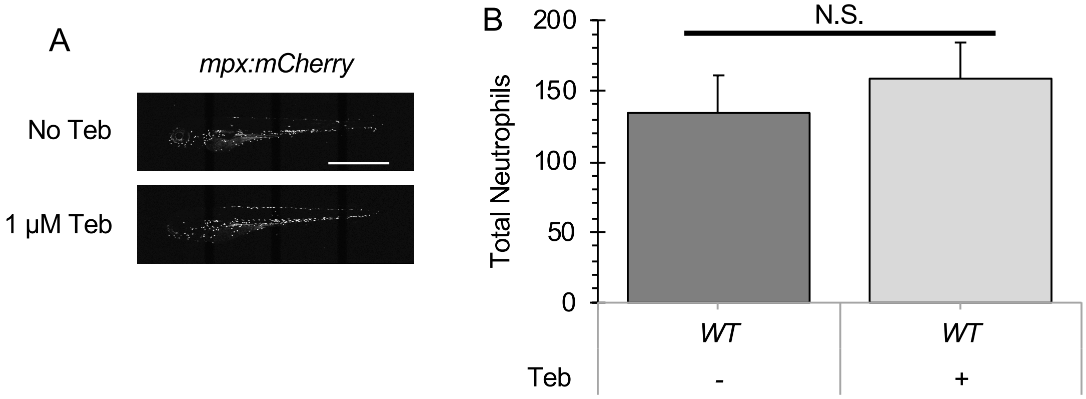

Supplemental Figure 4. Lack of neutrophil recruitment in Teb treated controls. **(A)** Representative confocal images of *mpx:mCherry* larvae. Shown here are larvae without Teb (No Teb) and with Teb treatment (+ 1 µM Teb). **(B)** Neutrophils were counted using FIJI Cell Counter and Total Neutrophil counts were plotted for each group. Error Bars are +1 standard deviation. Asterisks indicate significant differences *p<0.05 **p<0.01 ***p<0.001; N.S., not significant; by one-way ANOVA followed by Tukey's HSD Test. Scale bar in (A) is 1 mm.
